# A chronological expression profile of gene activity during embryonic mouse brain development

**DOI:** 10.1007/s00335-013-9486-7

**Published:** 2013-11-19

**Authors:** P. Goggolidou, S. Soneji, N. Powles-Glover, D. Williams, S. Sethi, D. Baban, M. M. Simon, I. Ragoussis, D. P. Norris

**Affiliations:** 1 Mammalian Genetics Unit, MRC Harwell, Harwell Science and Innovation Campus, Oxfordshire, OX11 0RD UK; 2 Weatherall Institute of Molecular Medicine, John Radcliffe Hospital, University of Oxford, Headington, Oxford, OX3 9DS UK; 3 Henry Wellcome Building of Genomic Medicine, Wellcome Trust Centre for Human Genetics, Roosevelt Drive, Oxford, OX3 7BN UK; 4 UCL Cancer Institute, Paul O’Gorman Building, University College London, 72 Huntley Street, London, WC1E 6DD UK; 5 Present Address: AstraZeneca, R&D, Innovative Medicines, 23F71 Mereside, Alderley Park, Macclesfield, Cheshire SK10 4TG UK

## Abstract

The brain is a functionally complex organ, the patterning and development of which are key to adult health. To help elucidate the genetic networks underlying mammalian brain patterning, we conducted detailed transcriptional profiling during embryonic development of the mouse brain. A total of 2,400 genes were identified as showing differential expression between three developmental stages. Analysis of the data identified nine gene clusters to demonstrate analogous expression profiles. A significant group of novel genes of as yet undiscovered biological function were detected as being potentially relevant to brain development and function, in addition to genes that have previously identified roles in the brain. Furthermore, analysis for genes that display asymmetric expression between the left and right brain hemispheres during development revealed 35 genes as putatively asymmetric from a combined data set. Our data constitute a valuable new resource for neuroscience and neurodevelopment, exposing possible functional associations between genes, including novel loci, and encouraging their further investigation in human neurological and behavioural disorders.

**Electronic supplementary material:**

The online version of this article (doi:10.1007/s00335-013-9486-7) contains supplementary material, which is available to authorized users.

## Introduction

The mammalian brain develops from a simple neuroepithelium into a complex and highly patterned organ showing distinct regionalisation, organisation, and cell-type specification. During this process, significant cell differentiation and tissue specialisation occur, cells migrate between regions, and neurons grow to make connections. Ultimately, this gives rise to the complex patterning and function that we see in the adult brain. Neuronal development can be thought of as comprising the stages of neurogenesis, neuron migration, axon outgrowth, and circuit formation, ultimately resulting in the functioning brain (Dixon-Salazar and Gleeson 2010; Kandell et al. 2000). These processes, which consist of complex molecular and cellular events, occur for each developing neuron, leading to the formation of functional neural circuits. Defects in these processes can underlie patterning, behavioural, and neuropsychiatric disorders (Arber 2012; den Heuvel et al. 2010; Hashimoto and Hibi 2012).

In the developing mouse embryo, simple head folds are first evident at about 7.5 days post coitum (dpc), quickly developing into the neural folds by 8.0 dpc. These become elevated and begin to fuse, ultimately completing fusion at the anterior neuropore at 9.5 dpc. The brain becomes further regionalised along the rostrocaudal axis, giving rise to a distinct fore-, mid-, and hindbrain. Over the next 1–2 days the wall of the developing brain thickens and by 11.0 dpc it comprises three layers: the inner ependymal, intermediate mantle, and outer marginal layers (Kaufman 1992). During this period the sensory structures the eye and the ear develop, resulting by 12.5 dpc in an evident lens and a fully formed otic vesicle (Kaufman 1992).

Development of the mammalian brain results in distinct left and right sides that display functional left–right (L–R) asymmetries. At the neuroanatomical level, such asymmetries are evidenced by variations in the shape and size of comparable regions, in subnuclear and cytoarchitectural organization of nuclei, in the level of neurotransmitter expression, and in cortical architecture (Hüsken and Carl 2012; Phillips and Thompson 2012; Yonehara et al. 2011). Normal L–R brain asymmetry in humans has been associated with behaviour, cognition, and emotion (Beraha et al. 2012; Lancaster et al. 2012), while abnormalities of cerebral asymmetry are associated with a number of disorders, including schizophrenia and autism (Knaus et al. 2012; Yan et al. 2012). While the basis of mammalian visceral L–R asymmetry has become well established (Hirokawa et al. 2009; Nakamura and Hamada 2012), little is known about how this originates in the brain and no connections have been made between visceral and brain L–R asymmetries in mammals (Mercola and Levin 2001; Norris 2012). High-resolution magnetic resonance imaging in male mice identified structural asymmetries in the medial-posterior regions of the thalamus, the cortex, and the hippocampus, with the left region being larger than the right in each case (Spring et al. 2010). These findings of asymmetric structures were not, however, associated with genetic asymmetries. A serial analysis of gene expression (SAGE) of human left and right embryonic hemispheres did identify ~100 putative L–R asymmetric loci at 12, 14, and 19 weeks of gestation (Sun et al. 2005). Lim domain only 4 (*LMO4*) demonstrated particularly strong variation; however, in the mouse a random asymmetry of *Lmo4* was detected, with different embryos showing either left or right dominant expression in 11.5- and 15.5-dpc cortex, suggesting that this does not underlie morphological asymmetry. No earlier L–R asymmetries of neural gene expression have been described and the mechanisms underlying this process remain unknown.

While many elements of neural development have become evident at the genetic level over the past decade, still more remains to be elucidated. The number of genes expressed within the brain is very high, as evidenced by the Allen Brain Atlas (www.brain-map.org), yet this serves to document expression in the adult brain rather than provide an insight into the expression that led to this organisation. Recent attempts to redress this in the Allen Developing Mouse Brain Atlas are as yet incomplete and comprise four embryonic stages (11.5, 13.5, 15.5, and 18.5 dpc) and currently only a limited set of genes (Henry and Hohmann 2012). To help elaborate the genetic processes underlying brain development, we have transcriptionally profiled mouse brains between 8.5 and 12.5 dpc, the period when neural progenitors shift from proliferation to neuronal differentiation. Furthermore, we have assessed expression of individual genes on the left and right sides of the developing brain. We present data showing 2,400 genes to be differentially expressed in the early stages of mouse brain development. These genes display varying expression profiles, reflecting diverse roles in the molecular and cellular mechanisms underpinning brain structure and function. Gross L–R expression differences were not obvious at any single stage; however, subtle putative differences may be reflected in the results of a stage-independent analysis. Our study provides novel information about differential gene expression in the developing brain, a complex organ linked to a great number of serious human conditions.

## Materials and methods

### Mouse lines and sample preparation

(C3H/HeH × 101/H)F_1_ embryos were dissected at 8.5, 10.5, and 12.5 dpc. Heads were dissected from the body and separated along the midline into left and right hemispheres. RNA was extracted using the Qiagen RNeasy kit (Qiagen, Valencia, CA, USA; catalog No. 74104) with DNAse I digestion according to the manufacturer’s instructions, quantified by NanoDrop 8000 (Thermo Scientific, Waltham, MA, USA), and quality assessed with the Agilent 2100 Bioanalyzer (Agilent Technologies, Santa Clara, CA, USA). cDNA was prepared using the High Capacity cDNA Synthesis kit (Applied Biosystems, Foster City, CA, USA).

Quality of hemisphere separation was assessed by quantitative reverse transcriptase polymerase chain reaction (qRT-PCR) analysis for the midline markers *Wnt1* (Mm01300555_g1) and *Shh* (Mm00436528_m1, Applied Biosystems). Correct dissection of the samples was confirmed by equivalent left and right *Wnt1* and *Shh* expression across the midline using β-actin as the reference gene; samples defined as correctly dissected were used for subsequent microarray hybridization. Twelve such validated sample pairs (4 per stage) were analysed for differential gene expression using Illumina microarrays (Illumina, San Diego, CA, USA).

### Microarray hybridization

RNA concentration was normalized to 50 ng/μl, and 11 μl was used to produce biotin-labelled complementary RNA (cRNA) using the Illumina^®^ TotalPrep™-96 RNA amplification kit (Ambion, Life Technologies, Carlsbad, CA, USA; catalog No. 4393543). Biotin-labelled cRNA of 1,500 ng from each sample was hybridized according to the Illumina whole-genome gene expression direct hybridization assay (Illumina, catalog No. 11286340) against the high-density Illumina mouse WG-6_V1.1_R1_11234304 oligonucleotide arrays, designed to detect 46,628 transcripts. The hybridized arrays were washed and then labelled with streptavidin-Cy3. Fluorescence emissions were quantitatively detected using BeadArray Scanner (Illumina) and analysed with BeadStudio software.

### Microarray analysis

The Illumina probe intensities were quantile normalised prior to differential expression using Linear Models for Microarray Data (LIMMA) (Smyth 2005). The model incorporated three factors: hemisphere, mouse, and stage from which the sample was extracted. Differentially expressed probes between stages were identified as those with *B* > 2 for all pairwise comparisons. Hemisphere-specific genes were those with *B* > 1. Statistically overrepresented molecular and biological processing Gene Ontology (GO) terms (http://www.geneontology.org/GO.format.obo-1_2.shtml#S.4) were found for different sets of genes using Database for Annotation, Visualization and Integrated Discovery (DAVID) (da Huang et al. 2009a, b) and the Biological Networks Gene Ontology tool (BINGO) ver. 2.44. The differentially expressed probes identified by LIMMA were *z*-score normalised to centralise and standardise each expression level; normalization is done so the Euclidean distance can be used to cluster. *z*-score normalisation was undertaken to standardise each expression profile over all samples (mean = 0, SD = 1). Subsequently, *k*-means clustering was carried out using the “kmeans” function in R; samples were clustered into nine partitions representing distinctly different expression patterns. The differentially expressed gene lists have been submitted in GEO and can be found at http://www.ncbi.nlm.nih.gov/geo/query/acc.cgi?acc=GSE44932.

### qRT-PCR analysis

Five micrograms of RNA was isolated from wild-type (C3H/HeH × 101/H) F_1_ embryonic heads at 8.5, 10.5, and 12.5 dpc using the RNeasy mini kit (Qiagen). cDNA was prepared for qRT-PCR using the High Capacity cDNA Reverse transcription kit (Applied Biosystems). qRT-PCR was performed in triplicate on six different heads at each developmental stage for the genes *Dcx* (Mm00438400_m1), *Myt1* (Mm00456190_m1), *Cryba2* (Mm00517617_m1), *Crybb1* (Mm00517828_m1), *Trh* (Mm01182425_g1), *Igdcc3* (Mm00501289_m1), *Slc2a3* (Mm00441483_m1), *Nr6a1* (Mm00599848_m1), *Myh7* (Mm01319006_g1), and *Myl3* (Mm00803032_m1). *Rps11* (Mm02601829_g1) and *β*-*actin* (Mm01205647_g1, Applied Biosystems) were used as the reference genes during the microarray validation and sample dissection validation, respectively. Alterations in gene expression were expressed relative to the mean intensity in 8.5-dpc heads, which were given a standardised value of 1. Negative controls of reactions without cDNA template were included. All reactions were conducted on the Applied Biosystems 7500 Fast Real-Time PCR system using the TaqMan Fast Universal PCR Master mix (catalog No. 4364103).

## Results

### A neuro-developmental expression profile in early brain development

In order to elucidate temporally changing gene expression during early to mid-gestation cranial development, 8.5-, 10.5-, and 12.5-dpc embryonic mouse heads were expression profiled by microarray. These were subdissected along the midline into left and right brain to further allow any L–R neural expression asymmetries to be assessed. The precision of L–R separation was confirmed by expression analysis of the midline markers *Wnt1* and *Shh*, as assessed by quantitative reverse transcriptase polymerase chain reaction (qRT-PCR). Only samples where left and right brain pairs showed equivalent expression for both loci (threshold difference less than 0.2-fold) were chosen for subsequent analysis, thereby minimising any bias for midline genes. Four sample pairs per embryonic stage were analysed for gene expression using the Illumina microarray platform. The resulting data were analysed for differential gene expression, using Linear Models for Microarray Data (LIMMA) (Smyth 2005). The analysis incorporated three factors: developmental stage, left versus right hemisphere, and the individual embryo. Differentially expressed genes were identified as those having a *B*-value (a measure of statistical significance) greater than 1, indicating statistical significance.

### Identification of gene clusters

**Fig. 1:**
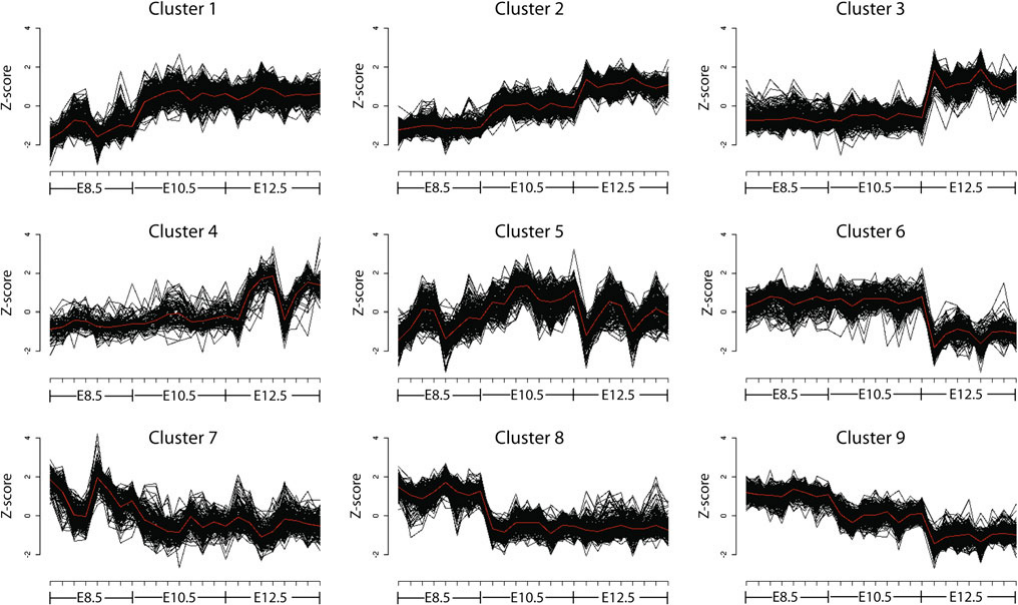
A total of 2,400 genes, differentially expressed at 8.5–12.5 dpc, were clustered into nine partitions according to their pattern of gene expression. *Clusters 1–4* represent increasing expression during development, *cluster 5* shows stable expression levels at the three developmental stages, and *clusters 6–9* correspond to decreasing gene expression across development. The gene expression pattern for each cluster is illustrated by a *thick red line* (Color figure online)

When gene expression analysis was conducted with respect to developmental stage, irrespective of hemisphere, a significant number of genes demonstrated expression changes. Exactly 2,400 genes were identified as showing a log of fold change (log_2_FC) greater than 2 and a *B* value greater than 2. Using *k*-means clustering, nine clusters were identified on the basis of similar patterns of changing gene expression (Fig. 1). Each cluster represents a unique trend during development. Clusters 1-4 represent increasing expression and clusters 6–9 represent decreasing expression (Fig. 1). Cluster 1 shows an increase in expression between 8.5 and 10.5 dpc and stable expression thereafter. Cluster 2 consists of genes that display an increase in their expression levels at every developmental stage. Clusters 3 and 4 (while clearly distinct) both contain genes with stable expression at 8.5 and 10.5 dpc but increasing at 12.5 dpc. In contrast, cluster 6 contains genes with stable expression at 8.5 and 10.5 dpc that decreases at 12.5 dpc. Clusters 7 and 8 are obviously distinct, yet both consist of genes that display a decrease in expression between 8.5 and 10.5 dpc and remain stable after that stage. Cluster 9 contains genes that display a gradual decrease in their expression at each developmental stage studied between 8.5 and 12.5 dpc. Strikingly, cluster 5 demonstrates relatively stable expression during development, when each stage is averaged; something the screen was not designed to do. There is, however, significant variation in the levels of gene expression within individual developmental stages, which explains why these genes were identified.

**Fig. 2:**
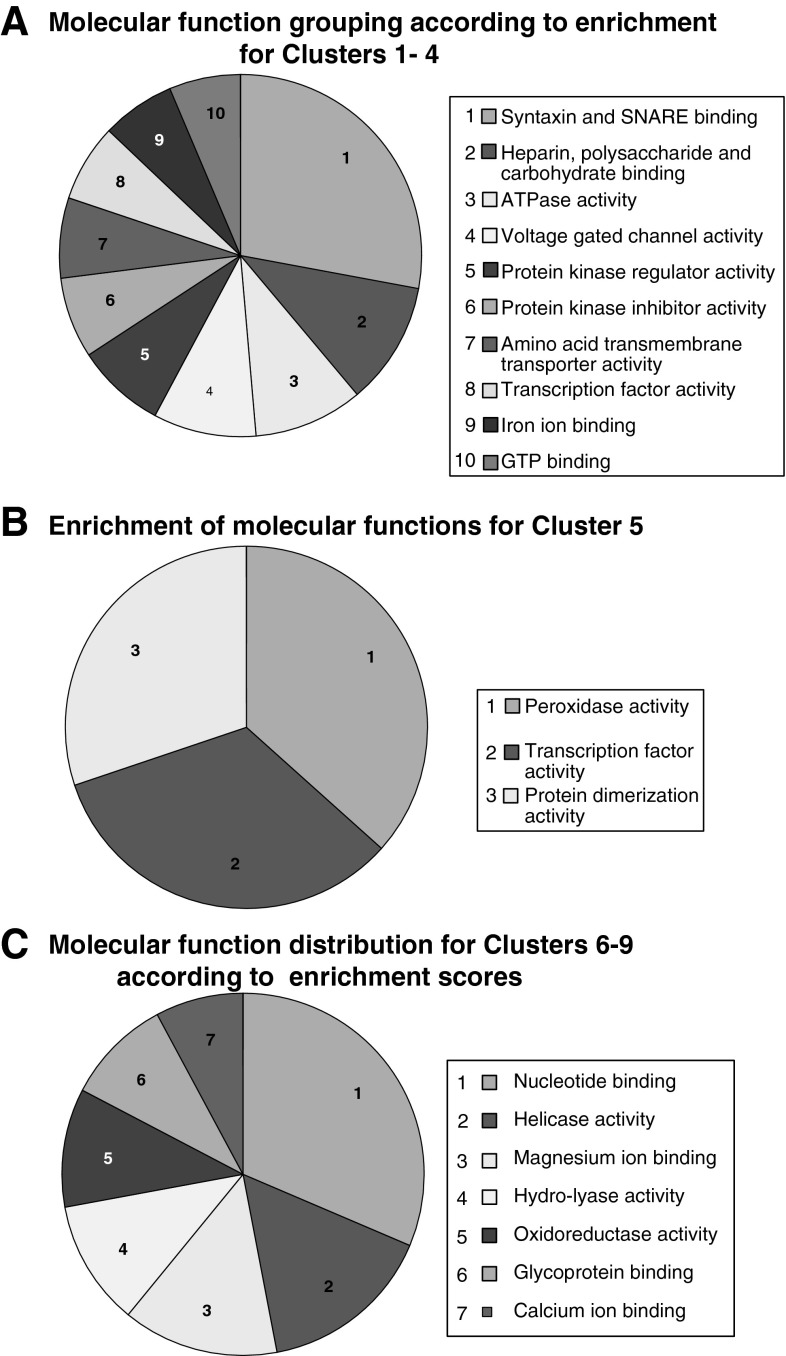
Representation of annotated molecular functions for clusters 1–9 according to enrichment scores. The investigation of molecular functions was conducted by DAVID; molecular functions are illustrated as *pie charts* according to their enrichment score. The greatest number of significant molecular functions was obtained for clusters 1–4 [10 clusters with enrichment scores ranging between 4.56 and 1.04 and *p* values ranging between 5.3E^−6^ and 9.6E^−2^ (**a**)], followed closely by clusters 6–9 (**c**). **b** Cluster 5, which included genes that showed stable levels of gene expression in the three developmental stages, consisted of three molecular functions with similarly significant enrichment scores

In order to further understand the nature of these gene clusters, gene ontology (GO) terms for biological processes and molecular functions were collected, initially for all nine clusters together (Supplementary Tables 1 and 2) and then, to allow for a more meaningful interpretation, for each cluster separately. Statistically overrepresented GO terms within each cluster were identified using the Database for Annotation, Visualization and Integrated Discovery (DAVID) (da Huang et al. 2009a, b). Cluster 5, the relatively stable expression cluster, comprises 144 genes (Fig. 1). Perhaps unsurprisingly, around two-thirds of these genes were associated with metabolic processes and one-third were relevant to biological regulation (Table 1). When we investigated possible molecular function through GO, we identified three molecular function GO terms with enrichment scores between 1.7 and 1.4. These contained peroxidase activity, transcription factor activity, and protein dimerization activity (Fig. 2b). Overexpression of glutathione peroxidase 1 is known to protect the developing mouse brain from hypoxic-ischemic injury (Autheman et al. 2012), reflecting the significance of peroxidase activity in brain development. Transcription factor activity, on the other hand, involves a number of key regulators of developmental processes, such as Otx2, which is required for the early specification of the brain (Gat-Yablonski 2011), or Neurog1/2, which coordinate the development of the olfactory system (Shaker et al. 2012).

**Table 1:** A total 2,400 differentially expressed genes grouped into nine clusters according to their expression patterns and described by their relevant GO terminology according to DAVID

GO term	GO term ID	No. genes	Gene examples
Cluster 1: 212 genes			
Anatomical structure development	GO:0048856	34	*Notch4*, *Wnt9a*, *Robo4*, *Dll1*, *Slit2*, *Sox8*
Cell fate commitment	GO:0045165	9	*Hes5*, *Dll1*, *Notch4*, *Tgfbr1*, *Sox8*, *Nr2e1*, *Foxn4*, *Ptf1a*, *Ascl1*
Inner-ear development	GO:0048839	4	*Hes5*, *Dll1*, *Muted*, *Pou3f4*
Metabolic process	GO:0008152	100	*Fads2*, *Nxn*, *Ccnc*, *Insig1*, *Glud1*
Gliogenesis	GO:0042063	3	*Slit2*, *Nr2e1*, *Ascl1*
Cell migration	GO:0016477	7	*Slit2*, *Sdcbp*, *Tgfbr1*, *Robo4*, *Unc5c*, *Nr2e1*, *Ascl1*
Genes not associated with GO terms		55	
Cluster 2: 343 genes			
Nervous system development	GO:0007399	39	*Neurog2*, *Kif5c*, *Sema4a*, ***Dcx***, *Gata2*, *Lhx8*
Neuron differentiation	GO:0030182	22	*Klf7*, *Myo6*, *Nrn1*, *Mapt*
Neurogenesis	GO:0022008	24	*Cntn2*, *Timp2*, *Stmn3*, *Stmn1*
Developmental process	GO:0032502	81	*Rb1*, *Isl1*, *Zic3*, ***Myt1***, *Sema6c*, *Cdkn1a*
Regulated secretory pathway	GO:0045055	11	*Syn1*, *Syt1*, *Cadps*, *Cplx1*
Cellular localization	GO:0051641	34	*Slc1a3*, *Hap1*, *Lin7a*, *Rab3d*
Vesicle-mediated transport	GO:0016192	24	*Snap25*, *Rab2*, *Stx7*, *Syp*
Central nervous system development	GO:0007417	12	*Lhx1*, *Lxh2*, *Lxh8*, *Gata2*, *Neurog2*
Genes not associated with GO terms		96	
Cluster 3: 260 genes			
Localization	GO:0051179	68	*Ephb1*, *Syt5*, *Scg2*, *Nrxn3*, *Tekt2*
Transport	GO:0006810	56	*Gria2*, *Slc1a1*, *Tubb4*, *Gdi1*
Synapse organisation and biogenesis	GO:0050808	9	*Nlgn3*, *Agrn*, *Nrxn1*, *Cacng2*
Cell projection organisation and biogenesis	GO:0030030	18	*Mt3*, *Tbr1*, *Gbx2*
Nervous system development	GO:0007399	26	*Chrd*, *Neurod2*, *Celsr3*
Axonogenesis	GO:0007409	12	*Reln*, *Chl1*, *Sema4f*, *Nfasc*
Ion transport	GO:0006811	24	*Camk2b*, *Cacna2d2*, *Kcnq2*, *Gria1*, *Atp6ap1*
Behaviour	GO:0007610	11	*Accn2*, *Gabrg2*, *Atp1a2*, *Mecp2*
Learning and/or memory	GO:0007611	5	*Accn2*, *Atp1a2*, *Amph*, *Gria1*, *Neurod2*
Genes not associated with GO terms		31	
Cluster 4: 78 genes			
Anatomical structure development	GO:0048856	32	*Cryba1*, *Pax1*, *Twist2*, *Anxa2*
System development	GO:0048731	28	*Eln*, *Actg2*, *Robo3*, *Anxa2*
Sensory organ development	GO:0007423	12	***Crybb1***, *Cryba4*, *Cryba1*, ***Cryba2***
Eye development	GO:0001654	6	*Lim2*, *Crygc*, *Crygs*, *Crygd*, *Mip*, *Mab21l2*
Genes not associated with GO terms		0	
Cluster 5: 144 genes			
Metabolic process	GO:0008152	78	*Taf12*, *Cdk4*, *Casp6*, *Ccnd1*, *Igf1*, *Evi1*, *Twist1*
Biological regulation	GO:0065007	45	*Tbx2*, *E2f2*, *Cdkn3*, *Cdk4*, *E130306D19Rik*
Genes not associated with GO terms		21	
Cluster 6: 129 genes			
Cell cycle	GO:0007049	25	*H2afx*, *Nde1*, *Gspt1*, *Ccne1*, *Fgf8*, *E2f3*
DNA-dependent DNA replication	GO:0006261	7	*Ccne2*, *Mcm2*, *Hus1*
Metabolic process	GO:0008152	76	*Tead2*, *Rad54l*, ***Igdcc3***, *Ipo8*, *Yap1*, ***Trh***, *Pdk3*
DNA repair	GO:0006281	8	*Fen1*, *Rad51ap1*, *Rfc5*, *Chaf1b*
Genes not associated with GO terms		13	
Cluster 7: 85 genes			
Amino acid metabolic process	GO:0006520	5	*Mat2a*, *Lars*, *Shmt2*, *Srr*, *Sdsl*
Tube morphogenesis	GO:0035239	5	*Tsc2*, *Pbx1*, *Nppb*, *Lmo4*, *Nppa*
Anatomical structure morphogenesis	GO:0009653	8	*Arnt*, *Tube1*, *S100a6*
Genes not associated with GO terms		67	
Cluster 8: 135 genes			
Organ morphogenesis	GO:0009887	17	*Tbx1*, *Wnt1*, *Hoxa2*, *Hoxb2*, *Gnas*
Anatomical structure development	GO:0048856	32	*Sufu*, ***Nr6a1***, *Sox10*, *Msh2*, ***Myh7***, *Nkx2*-*3*, ***Myl3***
Cell differentiation	GO:0030154	26	*Casp9*, *En1*, *Egfl7*
Skeletal morphogenesis	GO:0048705	4	*Tbx1*, *Hoxa2*, *Gnas*, *Tcfap2a*
Ear morphogenesis	GO:0042471	4	*Tbx1*, *Hoxa2*, *Wnt1*, *Edn1*
Anterior/posterior pattern formation	GO:0009952	4	*Tbx1*, *Wnt1*, *En1*, *Ifitm1*
Cell motility	GO:0006928	9	*Tbx1*, *Pten*, *Itga3*, *Enah*, *Nisch*, *Egfl7*, *Msh2*, *Podxl*, *Lama5*
Muscle development	GO:0007517	8	*Tbx1*, ***Myh7***, *Acta1*, *Pten*, ***Myl3***, *Actc1*, *Tnnt2*, *Lama5*, *Mef2c*
Blood vessel morphogenesis	GO:0048514	6	*Tbx1*, *C1galt1*, *Pten*, *Edn1*, *Egfl7*, *Mef2c*
Neural crest cell development	GO:0014032	3	*t*-*box 1*, *endothelin 1*, *laminin*, *alpha 5*
Genes not associated with GO terms		22	
Cluster 9: 196 genes			
RNA processing	GO:0006396	13	*Pa2g4*, *Exosc5*, *6720458F09Rik*, *2510012J08Rik*, *Srpk1*
Glucose metabolic process	GO:0006006	8	*Pkm2*, *Hk2*, *Gpd2*, *Gpi1*, ***Slc2a3***, *Pdk1*, *Tpi1*, *Eno3*, *Pfkl*
DNA repair	GO:0006281	9	*Pold1*, *Fancd2*, *Supt16h*, *Fanca*, *Mutyh, Brca2*, *Pole*, *Ruvbl2*, *Lig3*
DNA replication	GO:0006260	7	*Gtpbp4*, *Mcm5*
Genes not associated with GO terms		159	

The number of genes as well as examples of representative genes are shown for each cluster. GO processes are described and indicated by their GO term ID number. The ten genes tested by qRT-PCR are highlighted in bold. The genes with the highest individual changes over the stages analysed are summarized in Supplementary Table 4

### Genes with increasing expression during development

Clusters 1–4 were associated with increasing gene expression. Cluster 1, consists of 212 genes that showed an increase in expression between 8.5 and 10.5 dpc, followed by stable expression. When the GO terms for biological processes were examined, terms associated with anatomical structure development, cell fate commitment, inner ear development, metabolic processes, gliogenesis, and cell migration were present (Table 1). Clearly changes in “inner ear development” gene expression fit with the known timing of ear development. Both gliogenesis and cell migration would be expected to change in line with the development of brain structures at these stages.

In contrast to the increase in expression between 8.5 and 10.5 dpc observed for cluster 1, the 343 genes in cluster 2 showed continuously increasing expression between stages. Such temporal expression changes would be consistent with the expected increase in more terminally differentiated, neuronal cell types. Indeed, GO term analysis identified nervous system development, neuronal differentiation, neurogenesis, and developmental processes as significant biological processes (Table 1). Regulated secretory pathways, cellular localization, and vesicle-mediated transport were also identified terms, arguing that these too relate to the increasing neuronal function and possibly to axonal growth and signalling at synapses. Furthermore, vesicle-mediated transport has been demonstrated to be important for the release of gliotransmitters into the extracellular space, enabling communication between astrocytes and neurons (Kreft et al. 2009). Indeed, all of the biological GO terms identified for this cluster would be predicted to increase in line with continuing development and specialisation of cells within the embryonic brain.

Cluster 3 comprises 260 genes that showed stable expression until 10.5 dpc and then increased by 12.5 dpc. Analysis of the genes affiliated with it revealed enriched biological process GO terms that included localization, transport, synapse organisation, and biogenesis. These all fit well with the concept of advancing neural development and differentiation which are expected in the 12.5-dpc embryo. Nervous system development, axonogenesis, and ion transport were also identified as biological processes associated with cluster 3. Indeed, signs of axonogenesis are known to be exhibited by 12.5 dpc by Purkinje cells in the developing mouse lateral cerebellum (Miyata et al. 2010).

Finally, the LIMMA *z*-score algorithm clearly identified cluster 4 as separate from cluster 3, although it too showed expression that increases at 12.5 dpc; noticeably there is variation in expression levels between individual 12.5-dpc samples in this cluster (Fig. 1). The cluster contains 74 genes that, when analysed in DAVID, identified enrichment for the biological process GO terms of anatomical structure development, system development, sensory organ development, and eye development (Table 1). These are GO terms distinct from those identified for cluster 3. It is easy to see how these GO terms could be explained with respect to the known changes in head structure and development that occur between 10.5 and 12.5 dpc, the stages when the development of most substructures in the head is fast progressing.

Following the analysis of the GO terms for biological processes, an analysis of the GO terms for molecular function of the increasing-expression clusters (1–4) was also undertaken. When analysed separately, none of the rising clusters revealed any statistically significant results (data not shown). However, when the clusters were considered as a whole, statistically significant molecular function GO terms were identified. The most significant molecular functions were syntaxin and SNARE binding (Fig. 2a), both of which play an essential role in vesicle-mediated transport. Syntaxin and SNARE proteins are concentrated near release sites along the presynaptic membrane, allowing neurotransmission along motor nerve terminals (Walter et al. 2011). Other significant molecular functions were heparin and carbohydrate binding, ATPase activity, ion channel activity, and protein kinase regulator activity (Fig. 2a). Heparin sulphate proteoglycans (a major component of extracellular matrix) are believed to impact axon guidance through their role as cofactors for other proteins (Ariga et al. 2010; Laabs et al. 2005) such as the fibroblast growth factors, which are required for normal cranial development (Sansom and Livesey 2009). In contrast, ATPase, ion channel, and protein kinase regulator activities are required for synaptogenesis and synapse function, consistent with the establishment of axonal function (Berg and Hoogenraad 2012; Kandell et al. 2000).

### Genes with decreasing expression during development

Four gene clusters (6–9) showed decreasing expression over developmental time (Fig. 1). The patterns of expression were distinct for each cluster and were linked to decreasing expression at one or more developmental stages. Cluster 6 comprises 129 genes that showed stable expression at 8.5 and 10.5 dpc and decreasing expression by 12.5 dpc. Analysis of the ontology of biological processes revealed the most highly represented GO terms to be metabolic processes, followed by cell cycle, DNA repair, and DNA-dependent DNA replication (Table 1). It seems plausible that this may relate to genes dropping out of the cell cycle and becoming quiescent as they differentiate into neurons.

The 85 genes in cluster 7 displayed decreasing but noisy expression profiles over development. This cluster showed a high representation of the biological function GO terms associated with amino acid metabolic processes as well as anatomical structure morphogenesis, and tube morphogenesis. While a reduction in amino acid metabolism may relate to cellular differentiation and subsequent reduced proliferation, it is not immediately obvious why anatomical structure morphogenesis and tube morphogenesis are decreasing.

In comparison to cluster 7, a total of 135 genes, whose expression decreased between 8.5 and 10.5 dpc but remained constant thereafter, are separately grouped into cluster 8. While a simple description of the two clusters makes them sound highly similar, the degree of variability in expression at 8.5 dpc is different between clusters 7 and 8 (Fig. 1). The majority of genes in cluster 8 maintain high expression at 8.5 dpc but decreasing at 10.5 dpc. In contrast, the expression of genes in cluster 7 at 8.5 dpc (and to some extent the other time points) demonstrates greater variability. Thus, genes grouped in cluster 7 are more stochastic throughout the developmental stages than those in cluster 8. This is similar to the situation described for cluster 4. Perhaps unsurprisingly, therefore, the biological function GO terms identified for cluster 8 were distinct from those classified in cluster 7 and included anatomical structure development, cell differentiation, organ morphogenesis, cell motility, and muscle development (Table 1). Although the apparent reduction in cell differentiation and organ morphogenesis between 8.5 and 10.5 dpc may seem counterintuitive, it is specific to a small number of genes. Importantly, the annotation of genes with GO terms is based on gene function in the whole organism. This raises the possibility of biological function of these genes varying between tissues.

Lastly, cluster 9 contains 196 genes with clearly decreasing expression across development. The biological functions of cluster 9 included RNA processing, DNA repair, glucose metabolic processes, and DNA replication (Table 1). It is possible that these changes relate to reduced cell proliferation and increased terminal cellular differentiation seen with developmental age.

Upon investigation of the molecular function GO terms for the genes present in the decreasing-expression clusters (6–9), no significant results were evident for individual clusters (data not shown). Seven groups, however, became evident when the decreasing-expression clusters were considered together; enrichment scores ranged from 5.53 to 1.38. The most significant molecular functions of these clusters were nucleotide binding, helicase activity, magnesium ion binding, and hydro-lyase activity (Fig. 2c). This progressive decrease in specific functions seems likely to be associated with changes in the distribution of cell types in the developing brain.

Finally, in order to further validate the GO terminology identified with the nine clusters through DAVID, an additional tool, called BINGO, which assesses overrepresentation of Gene Ontology categories, was also used. Our initial GO terminology was thus validated as there was great overlap in the identified Gene Ontology categories between the two methods (data not shown).

### qRT-PCR-based validation of microarray data

**Fig. 3:**
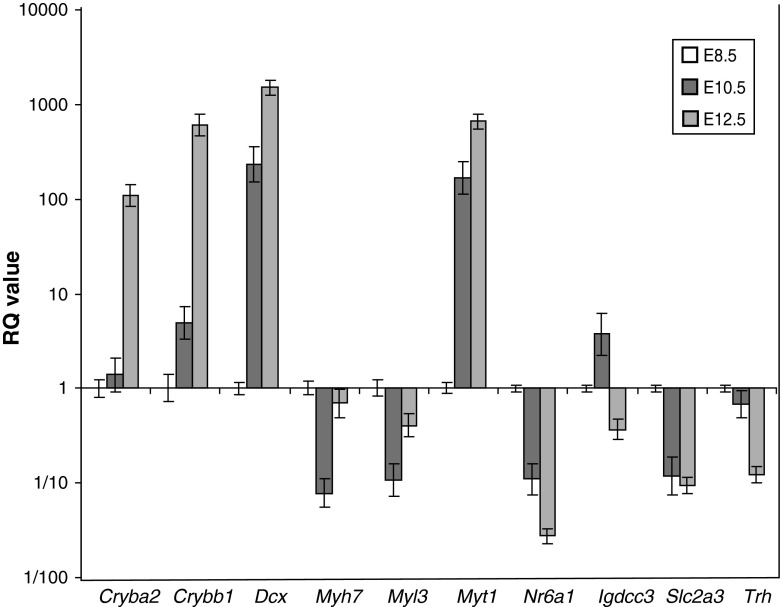
Differential gene expression patterns during mouse embryonic head development validated by qRT-PCR. Ten genes, each representative of the nine clusters, were tested for expression at 8.5, 10.5, and 12.5 dpc. Gene expression was normalized relative to 8.5 dpc and was given a relative quantification (RQ) value of 1. *Rps11* was the endogenous control and six biological replicates, with three technical replicates each, were performed at each stage, with the results displayed on a logarithmic scale

To confirm elements of the microarray dataset, ten loci were chosen from the nine clusters for validation by qRT-PCR (Fig. 3). For this analysis an additional 18 embryonic heads were collected and dissected into left and right sides, as was done for the original analysis; six per developmental stage at 8.5, 10.5, and 12.5 dpc. RNA was produced and quality controlled as before. To control for sample variation, *Rps11* was chosen as the endogenous control that displayed the least variance in the microarray dataset as well as a high-level mean expression (data not shown).

The genes chosen for validation had been clustered into one of the nine clusters, representing increasing or decreasing expression across the three developmental stages. The expression profiles obtained by qRT-PCR were consistent with the expression data acquired from the microarray for most of the investigated genes. Consistency between the microarray and qRT-PCR data was observed for the *Dcx* (cluster 2), *Myt1* (cluster 2), and *Cryba2* (cluster 4) genes, representing increasing expression during development (Fig. 3). Similarly compatible results were obtained for *Trh* (cluster 6), *Igdcc3* (cluster 6), *Myl3* (cluster 8), *Nr6a1* (cluster 8), and *Slc2a3* (cluster 9), validating the decreasing expression profiles observed in the microarray.

During qRT-PCR validation, two genes whose qRT-PCR expression did not correspond to the microarray expression were identified. Although showing an increase in expression only at 12.5 dpc in the microarray dataset, *Crybb1* displayed a gradual increase in expression during development by qRT-PCR, which shows that the analysis failed to identify the low-level increase between 8.5 and 10.5 dpc. qRT-PCR analysis of the cardiac marker *Myh7* (cluster 8) demonstrated a decrease in expression at 10.5 dpc, which returned to the 8.5-dpc expression level at 12.5 dpc. Intriguingly, two different probes were present in the microarray for the detection of *Myh7* expression. The expression pattern of one of them closely resembled the *Myh7* qRT-PCR expression profile, whereas the other probe detected lower levels of comparable expression across all three developmental stages. It seems most likely that this relates to differing efficiencies of the two microarray probes, although we cannot rule out the presence of different splice isoforms.

### Analysis of differential gene expression between left and right hemispheres

Visceral L–R asymmetry in mouse is prefigured by asymmetric gene expression patterns that are evident from 8.5 dpc; this asymmetry in gene expression is maintained into organogenesis. While clear L–R differences occur in the anatomy and function of the mammalian brain hemispheres, only asymmetry of *Lmo4* has been reported and it occurs relatively late during development. Moreover, *Lmo4* asymmetry is random. It therefore seems reasonable to hypothesise that earlier asymmetries of gene expression exist. When left- and right-sided brain microarray results were compared at each developmental stage, no genes showed significant expression differences. However, when the data from all three developmental stages were combined, 35 genes demonstrated a *B* value of greater than 1 (Table 2).

**Table 2:** A total of 35 genes showed asymmetric gene expression between the left and right hemispheres across the three developmental stages

Gene ID	Accession no.	Log_2_FC	*B* value	*p* value	Adj. *p* value
*Pa2g4* ^a^	NM_011119	0.246048524	3.626402	3.65E−06	0.086542
***Ptma*** ^a^	NM_008972.1	−0.445023482	3.61533	3.71E−06	0.086542
*2500002G23Rik* ^a^	XM_289903	0.347354334	3.120577	7.53E−06	0.110588
*2310007O11Rik* ^a^		0.275456441	2.716847	1.32E−05	0.110588
*Egfl4* ^a^	XM_194337	0.309514961	2.71044	1.33E−05	0.110588
*Trrp2* ^a^	AK018463	0.206433383	2.397186	2.05E−05	0.110588
*1110002E23Rik* ^a^	AK003291	0.298272881	2.38787	2.07E−05	0.110588
*2610528H13Rik*	NM_145944	0.206048351	2.377478	2.10E−05	0.110588
*Rps6*	NM_009096.1	−0.291050752	2.367105	2.13E−05	0.110588
***Arpc4***	AK030840	0.199773095	2.264929	2.45E−05	0.114277
*LOC216443*	XM_125952.4	0.215562754	2.092713	3.09E−05	0.121441
*2610028H07Rik*	AK011590	0.238605224	2.059119	3.23E−05	0.121441
*Tuba1* ^a^	NM_011653	−0.29198816	1.977735	3.60E−05	0.121441
*H3f3a* ^a^	NM_008210.2	−0.404992731	1.96844	3.64E−05	0.121441
*Fbxo3*	NM_212433.1	−0.212311389	1.79953	4.56E−05	0.137831
*C330034C07Rik*	AK082825	0.313192465	1.772177	4.73E−05	0.137831
*2700083E18Rik*		0.271117214	1.655589	5.51E−05	0.144869
*B930085B11Rik*	AK081092	0.14489787	1.632832	5.68E−05	0.144869
	AK010224.1	0.296980821	1.520376	6.58E−05	0.144869
*2900092E17Rik*	NM_030240.1	0.171958701	1.515811	6.62E−05	0.144869
	AK088505.1	0.525904741	1.495879	6.79E−05	0.144869
*Hist1h2ah*	NM_175659.1	0.174386481	1.458561	7.13E−05	0.144869
*Rapgef1*	NM_054050	0.168088916	1.43871	7.32E−05	0.144869
***Btf3*** ^a^	NM_145455.1	0.259158405	1.424814	7.45E−05	0.144869
*5730441M17Rik*	AK017632	0.190825576	1.3412	8.31E−05	0.155038
*1110007M04Rik*	NM_026742.1	−0.289482866	1.199129	9.98E−05	0.16287
*1110036I07Rik*		0.171935148	1.183364	0.000102	0.16287
*Scamp5*	NM_020270.2	0.217801578	1.176375	0.000103	0.16287
*Sf3a1*	NM_026175	0.121273306	1.136752	0.000108	0.16287
*Hmgb1*	NM_010439.2	−0.256773927	1.13633	0.000108	0.16287
*Hist1h2ao*	NM_178185.1	0.145863469	1.082718	0.000116	0.16287
***Sf3b2***	NM_030109.1	0.213756797	1.079379	0.000116	0.16287
*Chd3*	NM_146019.1	0.309509325	1.058934	0.00012	0.16287
*Fkbp5*	NM_010220.2	0.229890096	1.02514	0.000125	0.16287
*Mapk6*	NM_015806.2	0.173487645	1.02027	0.000126	0.16287

log_2_FC is the log of fold change between the left and right hemispheres normalized for the right hemisphere. The statistically significant *B*, *P* and adj. *P* values for each gene are also clearly indicated. The four asymmetrically expressed genes also identified by Sun et al. (2005) are highlighted in* bold*

^a^The ten genes tested by real-time PCR for left–right differences

The log_2_FC for these genes ranged from −0.44 to 0.35 (~0.74-fold to ~1.3-fold expression changes, respectively); the conventionally used cutoff value of 1 corresponds to a twofold expression change. Such values can reflect either experimental noise or true but small differences in gene expression. To examine these possibilities, we used qRT-PCR to analyse the expression of the ten genes with the highest *B* or log_2_FC values (Table 2). RNA from the left and right sides of eighteen 8.5-, 10.5-, and 12.5-dpc embryos (6 embryos per stage) was analysed and three technical replicates and two repeats of each assay were performed (data not shown). This failed to reveal any consistent L–R expression differences.

**Fig. 4:**
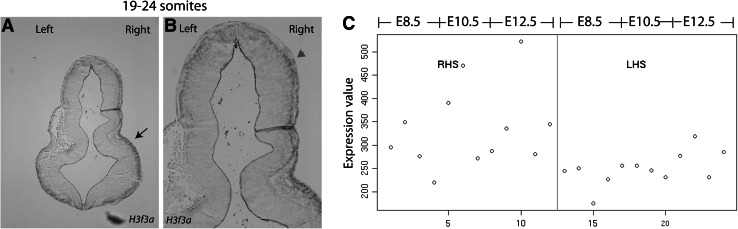
*H3f3a* displayed asymmetric gene expression between the left and right brain hemispheres across development. More cephalic mesenchymal (**a**) and neuroepithelial cells (**b**) expressed *H3f3a* on the right hemisphere at 19–24 somites, consistent with the expression pattern detected by microarray analysis (**c**)

One gene with significant differences in expression between the left and right hemispheres was chosen for validation by in situ hybridisation. *H3f3a* displayed greater expression in the right hemisphere, with a log_2_FC of 0.4 and a *B* value of 1.97 (Table 2). In situ hybridisation analysis demonstrated a slightly greater expression in the right-hand side of the brain at 19–24 somites (Fig. 4a). Specifically, more neuroepithelial (red arrowhead, Fig. 4b) and cephalic mesenchymal cells (black arrow, Fig. 4a) expressed *H3f3a* in the right hemisphere, mimicking the expression detected in the microarray (Fig. 4C). However, the difference in the number of cells expressing *H3f3a* between the left and right hemispheres was small, making subsequent investigation challenging.

**Fig. 5:**
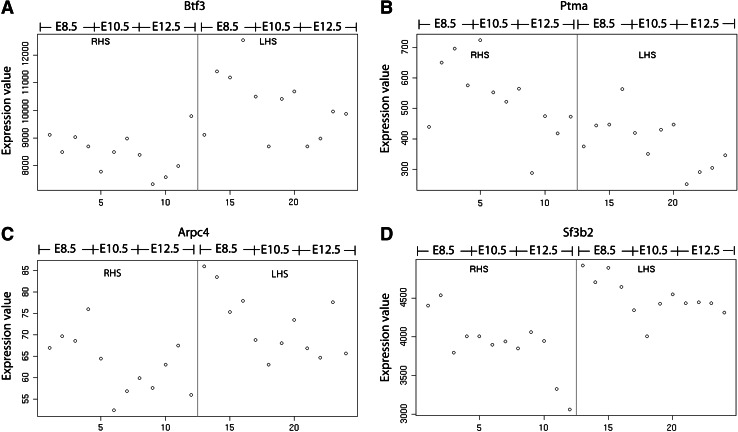
Four genes were identified as putatively differentially expressed between the two mouse embryonic hemispheres at 8.5–12.5 dpc. **a** *Btf3* displayed higher levels of expression in the left hemisphere at 8.5, 10.5, and 12.5 dpc. **b** *Ptma* was more highly expressed in the right hemisphere for the three developmental stages. **c** *Arpc4* was noticeably more expressed in the left hemisphere, with the difference in expression between the two hemispheres most obvious at 8.5 dpc. **d** *Sf3b2* displayed an overall trend of higher expression in the left hemisphere, most apparent at 10.5 dpc. The white dots represent the normalized expression levels of each sample for the specific gene. *RHS* right hemisphere, *LHS* left hemisphere

Our Illumina chip contained two probes for *Lmo4*, but neither revealed significant L–R expression differences (data not shown). However, 4 of the ~100 additional loci implicated by Sun et al. (2005) were identified by our screen (Table 2, indicated in bold). *Btf3* displayed asymmetric expression in our dataset, with a log_2_FC of 0.26 between the left and right hemispheres, consistent with stronger expression in the left hemisphere (Fig. 5). *Ptma* was more strongly represented in the mouse right hemisphere, with a log_2_FC of −0.44. Furthermore, we found higher levels of both *Arpc4* and *Sf3b2* in the mouse left hemisphere (log_2_FC of 0.2 and 0.21, respectively). Comparison of our data with that of Sun et al. (2005) for the above four genes revealed consistency in the sidedness of differential gene expression (Supplementary Table 3). This overlap between the datasets suggests that although challenging to validate, some of the loci that we have identified might show legitimate L–R expression differences and could also demonstrate concordance in differential gene expression between rodents and humans.

## Discussion

The adult brain is an intricate and exquisitely patterned organ. While great strides in understanding its function have been made, its development from a simple epithelium into the adult brain remains far from understood. In this study we have concentrated on the early events associated with its initial patterning, by expression profiling 8.5-12.5-dpc head development. This has identified 2,400 genes as being differentially expressed; 1,300 displayed increasing expression with developmental age, suggestive of function associated with complexity. Comparison of biological process GO terms within individual clusters showed that genes with similar function exhibit similar variations in temporal expression. These genes were involved mainly in development, transport, and cell localization. One example was the high expression of crystallins seen by 12.5 dpc. Crystallins are expressed in the lens of the adult eye (Andley 2007), and we showed that this expression starts early in development, before the eye has properly formed.

Approximately 900 genes showed a decreasing expression pattern during development, such that their expression decreased between 8.5 and 12.5 dpc. The gene ontology terms for these genes included metabolic processes, the cell cycle, DNA repair, certain aspects of development and morphogenesis, and RNA processing; these are expected to slow down as the brain differentiates into the more specialized substructures. Finally, around 200 genes displayed an unchanged expression pattern during development; they were involved in metabolic processes and biological regulation. Clearly as these genes were identified, their expression cannot be truly unchanging. Indeed, a visual inspection of the results as displayed in Fig. 1 reveals levels of variation between samples, similar to that seen in other clusters. However, this variation occurs within, rather than between, developmental stages. A number of possible explanations exist: (1) These genes show strong expression variation over short time scales. The innate variability of the speed of embryogenesis, even within single litters, means that samples collected at a single time point in fact represent a small range of developmental ages. (2) The reliability of dissection of the head from the body was imperfect, leading to small variations in the tissue analysed between samples. Genes expressed in the tissue that was in only some samples would then vary significantly. (3) There was low-level gene expression in combination with experimental noise. To what extent this reflects the overall false-positive rate is difficult to know.

While the descriptions of clusters 3 and 4 seem identical, comparison of the *z*-scores for each sample shows clearly changing levels of expression. The cluster 4 gene expression at 12.5 dpc shows significant variability, with a clear downward “spike” evident for one sample (Fig. 1). Similarly, clusters 7 and 8 are far from identical, with cluster 7 loci showing very distinct sample-to-sample variations within the 8.5-dpc results. The likely explanations for these variations presumably overlap the reasons already proposed for cluster 5.

Overall, the microarray dataset fits in with the already known processes that take place in the head, validating the quality of our analysis. Furthermore, previously characterized genes that have not been associated with the brain in the past display a differential expression profile in our microarray analysis; investigation for their putative involvement in brain development may prove fruitful. The novel genes included in our nine clusters, whose biological function remains unknown, also present valid candidates in the biological processes that take place in the head and brain. The data that we present here provide a resource openly available to researchers. Intriguingly, Hartl et al. (2008) have similarly examined differential gene expression in the heads of C3H embryos between 9.5 and 13.5 dpc. The two studies examined overlapping developmental stages but in different mouse strains and using different microarray platforms. While we observed some functional overlap in the gene ontology terms that we have identified, Hartl and colleagues showed the majority of differentially expressed genes to be involved in metabolic pathways, while our data has identified a range of anatomical, neuronal, morphogenesis, cell cycle, and metabolic processes (Table 2).

While human L–R neuroanatomical asymmetries are well characterised, in the mouse they have proved challenging to detect. A recent MRI-based study has clearly demonstrated reproducible L–R neuroanatomical asymmetry of mouse brains (Spring et al. 2010). While the pathways underlying visceral L–R asymmetry have been well studied and involve asymmetrically expressed master loci (López-Gracia and Ros 2007), little is known about how mammalian neural asymmetry is established; the only characterised asymmetric brain locus in mammals is *LMO4*, which shows more extensive right- than left-sided expression in humans (Sun et al. 2005). Intriguingly, random asymmetry of *Lmo4* expression was detected in mouse, suggesting that *Lmo4* asymmetry cannot underlie neuroanatomical asymmetry. Additional loci were identified in that study as being asymmetrically expressed and it remains possible that one or more of these might influence neural asymmetry.

Our experiments failed to detect any L–R asymmetry of gene expression at individual developmental stages. Both we and others have previously detected L–R asymmetry of loci impacting visceral asymmetry, demonstrating that such approaches are feasible (Hou et al. 2004; Stevens et al. 2010). In the light of such studies, the absence of any results at individual developmental stages argues against there being a simple strongly asymmetrically expressed neural locus in the mode of *Nodal* and *Pitx2*. However, it does not rule out transient asymmetries of gene expression that happen at stages we did not examine (perhaps a half-day or 1-day offset from the stages we have examined). In the case of visceral asymmetry, *Nodal* is asymmetrically expressed for only 6 h, although *Pitx2* asymmetry is maintained for at least 2 days. We must also consider the power of the study and its relationship to the number of cells demonstrating any asymmetry of gene expression. It may be that analysis of a larger set of samples at individual stages would identify asymmetric gene expression. In addition, it is not inconceivable that asymmetric expression of a locus could exist in parallel with symmetric expression in nearby tissues, thereby diluting apparent asymmetries of gene expression. Finally, we cannot exclude the possibility that probes designed to identify the genes that are asymmetrically expressed do not hybridise efficiently, or even that the genes fail to be represented on the chip.

The combined dataset of all stages analysed revealed apparent low-level L–R expression differences. These differences could not easily be validated; however, four of the loci had also been identified by Sun et al. (2005), and importantly they reported the same direction of L–R asymmetry as indicated by our data. In combination, our experiences suggest a number of considerations to be taken into account in future studies. It is important to realise that perhaps only a few cells in specific areas of the brain might be responsible for the observed asymmetric pattern of gene expression. In this case, it could be advantageous to isolate specific areas of the mouse brain, analysing solely L–R asymmetry of gene expression within a single brain domain; our experiments to date have not identified where these regions might be. Physical dissection of embryonic brain regions is clearly challenging, so the power of cell sorting could be harnessed to isolate labelled cells from the two brain hemispheres. The inclusion of different or more developmental stages, in combination with precise somite staging of embryos (providing 2-h developmental staging), may add to the analysis; however, at present it is impossible to guess which stages would be most appropriate. The use of next-generation RNA sequencing might prove more effective as this would allow increasing sequencing depth to be analysed for individual sample pairs as well as blindly identifying all transcripts, including splice variants, micro-RNAs, and even loci that have not been annotated. However, the added variation of library construction would be introduced. Finally, determination of the stage at which brain asymmetry is first evident in the mouse would provide a clear stage prior to which asymmetry must be being established; at present no physical asymmetry of embryonic or even neonatal mouse brains has been described.

The dataset that is presented here, combined with its validation, provides a valuable novel resource for researchers interested in neurodevelopment and brain function. It may prove of particular use to those studying brain development, suggesting novel gene associations, encouraging currently uncharacterised loci to be investigated, and promoting examination of their role in both brain development and function. Indeed, it seems likely that many of the loci we have identified have roles in human neurological function and behaviour.

## Electronic supplementary material

Below is the link to the electronic supplementary material.

Supplementary material 1 (DOCX 22 kb)
