# Supplementary material for: A chronological expression profile of gene activity during embryonic mouse brain development
Source: Mamm Genome. 2013 Nov 19;24(11):459–72. doi: 10.1007/s00335-013-9486-7 (PMC3843766; doi:10.1007/s00335-013-9486-7)
Supplement: Supplementary file 1 — Supplementary material 1 (DOCX 22 kb) [file 335_2013_9486_MOESM1_ESM.docx]

| **GO term** | **Description** | [**P-value**](http://cbl-gorilla.cs.technion.ac.il/GOrilla/6n5tc1ic/GOResults.html#p_value_info) |
| --- | --- | --- |
| [GO:0055114](http://www.godatabase.org/cgi-bin/amigo/go.cgi?query=GO:0055114&view=details) | oxidation-reduction process | 1.82E-08 |
| [GO:0007268](http://www.godatabase.org/cgi-bin/amigo/go.cgi?query=GO:0007268&view=details) | synaptic transmission | 3.44E-06 |
| [GO:0007399](http://www.godatabase.org/cgi-bin/amigo/go.cgi?query=GO:0007399&view=details) | nervous system development | 2.17E-05 |
| [GO:0032787](http://www.godatabase.org/cgi-bin/amigo/go.cgi?query=GO:0032787&view=details) | monocarboxylic acid metabolic process | 5.13E-05 |
| [GO:0001505](http://www.godatabase.org/cgi-bin/amigo/go.cgi?query=GO:0001505&view=details) | regulation of neurotransmitter levels | 7.01E-05 |
| [GO:0006695](http://www.godatabase.org/cgi-bin/amigo/go.cgi?query=GO:0006695&view=details) | cholesterol biosynthetic process | 7.43E-05 |
| [GO:0016126](http://www.godatabase.org/cgi-bin/amigo/go.cgi?query=GO:0016126&view=details) | sterol biosynthetic process | 7.43E-05 |
| [GO:0006629](http://www.godatabase.org/cgi-bin/amigo/go.cgi?query=GO:0006629&view=details) | lipid metabolic process | 1.46E-04 |
| [GO:0007409](http://www.godatabase.org/cgi-bin/amigo/go.cgi?query=GO:0007409&view=details) | axonogenesis | 1.57E-04 |
| [GO:0051969](http://www.godatabase.org/cgi-bin/amigo/go.cgi?query=GO:0051969&view=details) | regulation of transmission of nerve impulse | 2.10E-04 |
| [GO:0006631](http://www.godatabase.org/cgi-bin/amigo/go.cgi?query=GO:0006631&view=details) | fatty acid metabolic process | 3.11E-04 |
| [GO:0031644](http://www.godatabase.org/cgi-bin/amigo/go.cgi?query=GO:0031644&view=details) | regulation of neurological system process | 3.12E-04 |
| [GO:0050804](http://www.godatabase.org/cgi-bin/amigo/go.cgi?query=GO:0050804&view=details) | regulation of synaptic transmission | 3.49E-04 |
| [GO:0022900](http://www.godatabase.org/cgi-bin/amigo/go.cgi?query=GO:0022900&view=details) | electron transport chain | 4.80E-04 |
| [GO:0044255](http://www.godatabase.org/cgi-bin/amigo/go.cgi?query=GO:0044255&view=details) | cellular lipid metabolic process | 5.95E-04 |
| [GO:0043029](http://www.godatabase.org/cgi-bin/amigo/go.cgi?query=GO:0043029&view=details) | T cell homeostasis | 7.16E-04 |
| [GO:0023052](http://www.godatabase.org/cgi-bin/amigo/go.cgi?query=GO:0023052&view=details) | signaling | 7.46E-04 |
| [GO:0048858](http://www.godatabase.org/cgi-bin/amigo/go.cgi?query=GO:0048858&view=details) | cell projection morphogenesis | 7.49E-04 |

Supp. table 1. Gene ontology terminology of the biological processes relevant to the genes identified in all nine clusters, shown to be differential expressed during mouse brain development. The columns represent the GO term identified, along with its description and relevant p value, indicating statistical significance.

| **GO term** | **Description** | [**P-value**](http://cbl-gorilla.cs.technion.ac.il/GOrilla/hxlac7l1/GOResults.html#p_value_info) |
| --- | --- | --- |
| [GO:0016491](http://www.godatabase.org/cgi-bin/amigo/go.cgi?query=GO:0016491&view=details) | oxidoreductase activity | 1.93E-10 |
| [GO:0016667](http://www.godatabase.org/cgi-bin/amigo/go.cgi?query=GO:0016667&view=details) | oxidoreductase activity, acting on a sulfur group of donors | 2.12E-04 |
| [GO:0050662](http://www.godatabase.org/cgi-bin/amigo/go.cgi?query=GO:0050662&view=details) | coenzyme binding | 9.65E-04 |

Supp. table 2. Gene ontology terminology of the molecular functions applicable to the 2400 genes identified as differentially expressed during brain development. The GO terms, along with their descriptions and corresponding p values are clearly indicated.

| **Gene name** | **Our data** | **Expression pattern in our dataset** | **Expression pattern in Sun *et al*. dataset** | **Consensus expression** |
| --- | --- | --- | --- | --- |
| *Arpc4* | logFC=0.26 | Higher on Left side | Higher on Right side early in development | Unclear |
| *Btf3* | logFC=0.26 | Higher on Left side | Higher on Left side | Higher on Left side |
| *Ptma* | logFC=-0.44 | Higher on Right side | Higher on Right side | Higher on Right side |
| *Sf3b2* | logFC=0.21 | Higher on Left side | Higher on Left side | Higher on Left side |

Supp. table 3. Comparison of the findings of our and the Sun *et al*. dataset reveal consistency in differential gene expression between human and mouse brains for the four differentially expressed genes detected in both datasets.

| **Gene ID** | **Accession no.** | **Log_2_FC** | **P value** | **adj P Value** |
| --- | --- | --- | --- | --- |
| Pa2g4 | NM_011119 | 7.189016 | 3.65E-06 | 0.08654174 |
| Ptma | NM_008972.1 | -7.17903 | 3.71E-06 | 0.08654174 |
| 2500002G23Rik | XM_289903 | 6.74737 | 7.53E-06 | 0.110588133 |
| 2310007O11Rik |  | 6.414623 | 1.32E-05 | 0.110588133 |
| Egfl4 | XM_194337 | 6.40947 | 1.33E-05 | 0.110588133 |
| Trrp2 | AK018463 | 6.162118 | 2.05E-05 | 0.110588133 |
| 1110002E23Rik | AK003291 | 6.154893 | 2.07E-05 | 0.110588133 |
| 2610528H13Rik | NM_145944 | 6.146843 | 2.10E-05 | 0.110588133 |
| Rps6 | NM_009096.1 | -6.13882 | 2.13E-05 | 0.110588133 |
| Arpc4 | AK030840 | 6.060238 | 2.45E-05 | 0.114277229 |
| LOC216443 | XM_125952.4 | 5.929712 | 3.09E-05 | 0.121440829 |
| 2610028H07Rik | AK011590 | 5.904522 | 3.23E-05 | 0.121440829 |
| Tuba1 | NM_011653 | -5.84385 | 3.60E-05 | 0.121440829 |
| H3f3a | NM_008210.2 | -5.83696 | 3.64E-05 | 0.121440829 |
| Fbxo3 | NM_212433.1 | -5.71272 | 4.56E-05 | 0.137830841 |
| C330034C07Rik | AK082825 | 5.6928 | 4.73E-05 | 0.137830841 |
| 2700083E18Rik |  | 5.608454 | 5.51E-05 | 0.144869003 |
| B930085B11Rik | AK081092 | 5.592099 | 5.68E-05 | 0.144869003 |
|  | AK010224.1 | 5.511783 | 6.58E-05 | 0.144869003 |
| 2900092E17Rik | NM_030240.1 | 5.50854 | 6.62E-05 | 0.144869003 |
|  | AK088505.1 | 5.494397 | 6.79E-05 | 0.144869003 |
| Hist1h2ah | NM_175659.1 | 5.467986 | 7.13E-05 | 0.144869003 |
| Rapgef1 | NM_054050 | 5.453972 | 7.32E-05 | 0.144869003 |
| Btf3 | NM_145455.1 | 5.444178 | 7.45E-05 | 0.144869003 |
| 5730441M17Rik | AK017632 | 5.385497 | 8.31E-05 | 0.155038401 |
| 1110007M04Rik | NM_026742.1 | -5.28676 | 9.98E-05 | 0.162869527 |
| 1110036I07Rik |  | 5.27588 | 0.000102 | 0.162869527 |
| Scamp5 | NM_020270.2 | 5.27106 | 0.000103 | 0.162869527 |
| Sf3a1 | NM_026175 | 5.243788 | 0.000108 | 0.162869527 |
| Hmgb1 | NM_010439.2 | -5.2435 | 0.000108 | 0.162869527 |
| Hist1h2ao | NM_178185.1 | 5.206742 | 0.000116 | 0.162869527 |
| Sf3b2 | NM_030109.1 | 5.204458 | 0.000116 | 0.162869527 |
| Chd3 | NM_146019.1 | 5.190487 | 0.00012 | 0.162869527 |
| Fkbp5 | NM_010220.2 | 5.167445 | 0.000125 | 0.162869527 |
| Mapk6 | NM_015806.2 | 5.16413 | 0.000126 | 0.162869527 |
| 2310051F07Rik |  | 5.149956 | 0.000129 | 0.162869527 |
| Nfyb | NM_010914 | 5.149636 | 0.000129 | 0.162869527 |
| 2010005O13Rik | NM_145512.2 | -5.12697 | 0.000135 | 0.16551581 |
| Rdbp | NM_138580 | 5.046571 | 0.000157 | 0.186008387 |
| Semcap2 | NM_016867.1 | -5.0383 | 0.000159 | 0.186008387 |
| Idh1 | NM_010497 | 4.996529 | 0.000173 | 0.196485137 |
| Dlgap4 | NM_146128 | 4.977094 | 0.000179 | 0.197778306 |
| Pik4ca | NM_001001983 | 4.953518 | 0.000187 | 0.197778306 |
| C130033B18Rik | AK048072 | 4.92526 | 0.000198 | 0.197778306 |
| Lemd2 | NM_146075.1 | 4.925166 | 0.000198 | 0.197778306 |
| 3110001P07Rik | AK013971 | 4.918969 | 0.0002 | 0.197778306 |
| A730081D07Rik |  | 4.917923 | 0.000201 | 0.197778306 |
| LOC383787 | XM_357250.1 | -4.9106 | 0.000203 | 0.197778306 |
| 8430411H09Rik | NM_027805.1 | -4.88259 | 0.000215 | 0.204435238 |
| Cryzl1 | NM_133679.1 | -4.8623 | 0.000223 | 0.204594882 |
| 2610510D13Rik | NM_146130.2 | -4.86135 | 0.000224 | 0.204594882 |
| D030026A21Rik | AK050850 | 4.82156 | 0.000241 | 0.206727593 |
| Usp14 | NM_021522.2 | -4.82108 | 0.000242 | 0.206727593 |
| 3002006F17Rik | AK028251 | 4.813028 | 0.000245 | 0.206727593 |

Supp. Table 4. **Summary of the 55 genes that displayed the greatest expression changes across the three developmental stages.** The gene names, accession numbers, log of fold change (log_2_FC) across the stages and statistically significant P and adj. P values for each accession number are clearly indicated.
